# PREPARING FOR A GOLDEN AGE? APPROACH AND AVOIDANCE MOTIVATION IN THE CONTEXT OF OLD-AGE PREPARATION

**DOI:** 10.1093/geroni/igac059.508

**Published:** 2022-12-20

**Authors:** Fiona Rupprecht, Jana Nikitin

**Affiliations:** University of Vienna, Vienna, Wien, Austria; University of Vienna, Vienna, Wien, Austria

## Abstract

When it comes to old-age preparation, individuals may be motivated by positive outcomes they wish to approach (e.g., social connectedness) or by negative outcomes they wish to avoid (e.g., loneliness). We expected approach motivation to be adaptive in younger ages, when resources and possibilities for old-age preparation should be plentiful. For older adults, whose resources and time for (continued) old-age preparation are limited, the maintenance- and loss-oriented perspective of avoidance motivation may however be the more adaptive one. Using data from 2054 individuals aged 18 to 96 years and representing five cultures, we adopted a domain-specific, cross-cultural, and age-differential perspective on our research question. Results indicate that individuals tend to be both approach- and avoidance-motivated when it comes to old-age preparation and confirm the age-differential adaptivity of approach and avoidance motivation in terms of both, actual preparatory behavior and psychological well-being.

